# Educational Inequalities in Life and Healthy Life Expectancies among the 50-Plus in Spain

**DOI:** 10.3390/ijerph17103558

**Published:** 2020-05-19

**Authors:** Aïda Solé-Auró, Unai Martín, Antía Domínguez Rodríguez

**Affiliations:** 1 DemoSoc Research Group, Department of Political and Social Sciences, Universitat Pompeu Fabra, 08005 Barcelona, Spain; 2 Social Determinants of Health and Demographic Change—Opik Departament of Sociology 2, University of the Basque Country/Euskal Herriko Unibertsitate (UPV/EHU), 48940 Leioa, Spain; unai.martin@ehu.eus (U.M.); nara_beta@hotmail.com (A.D.R.)

**Keywords:** health inequalities, educational inequalities, life expectancy, healthy life expectancy, Spain, old people

## Abstract

This study computes educational inequalities in life expectancy (LE), healthy life expectancy (HLE), and unhealthy life expectancy (ULE) by gender and education level in Spain in 2012. Death registrations and vital status by level of education were obtained from Spain’s National Institute of Statistics. Health prevalences were estimated from the National Health Survey for Spain. We used Sullivan’s method to compute HLE, ULE, and the proportion of time lived with health problems. Our results reveal that Spanish women live longer than men in all education groups, but a higher proportion of women report poor health. We detect substantial differences in unhealthy life by gender and education, with higher effect for women and for those with low levels of education. Poor self-perceived health shows the largest educational gradient; chronic diseases present the lowest. This is the first work that provides evidence on health inequalities by education level in Spain. Our findings seem to be in line with reports of the smaller social inequalities experienced in Southern Europe and highlight the importance of education level on extending the proportion of years spent in good health in a Mediterranean country.

## 1. Introduction

Great advances in life expectancy (LE) have been recorded in most high-income countries over the last century, reflecting improvements in medical technology, better socioeconomic conditions, and healthier lifestyles. However, the fact that these advances are not necessarily accompanied by improvements in the population’s health has generated considerable debate. In addition to monitoring progress in the health of individuals, scholars have sought to determine if increasing longevity is associated with sustained country differences in health, particularly at older ages [1]. Equally, scholars stress the need to monitor gender and educational inequalities in longevity within populations, since understanding the associated mechanisms could help reduce inequalities between groups and improve a population’s overall health.

In Spain, any understanding of the social inequalities associated with healthy life expectancy (HLE) is scant. Recent studies have examined inequalities in LE [2,3], but only a few have analyzed HLE and mostly at a regional level [4,5] or in specific populations [6].

This study examines how the level of education limits progress in extending (H)LE at ages 50-plus by combining measures of length (mortality) and quality of life (morbidity) for Spanish men and women. Given that increasing our healthy lifespan is a universal goal, we expect that achieving a healthier society will reduce pressure on the public health system and that identifying the most deprived groups (probably those with the lowest level of education level) will help policymakers design appropriate plans of action. Additionally, to the best of our knowledge, this is the first study to estimate HLE in relation to the education level by sex at ages 50-plus in Spain, as until recently the data for the number of deaths by level of education were not publicly available.

### 1.1. Educational Inequalities in Life Expectancy

Over the last decade, European LE has increased by more than two years for both men and women [7], with women consistently outliving men in the majority of high-income countries [8]. The situation in Spain is particularly interesting given that (together with Switzerland) it leads the way in Europe in terms of having the highest LE at birth. Thus, for instance, Spanish LE at the age of 50 has increased, respectively, for men and women from 29.1 and 34.6 years in 2002 to 31.7 and 36.7 years in 2016 (latest data available) [7]. However, these remarkable increases in LE over the last half century mask marked disparities among population groups. For instance, in the United States, remaining LE at age 25 is about a decade shorter for those who do not have a high school degree compared to those who have graduated from college [9] (Figure 32). Jasilionis and Shkolnikov [10] provide an international overview on longevity across educational groups and conclude that those with high levels of education generally have the highest LE and, moreover, are the “vanguards” leading the way towards a lengthening of life for the remaining population groups. In short, educational attainment appears to be a very important indicator of an individual’s prospects for long life.

The case of Spain is somewhat unique, given that until very recently studies on socioeconomic differences in mortality remained unavailable, making it impossible to compute (H)LE by education level nationally. In 2018, however, Permanyer et al. [3] studied the evolution of LE over the last 50 years in Spain across four educational attainment groups. They reported that while LE was increasing for all education groups, they found evidence of increasing education gradients, particularly among men. However, little is known about HLE by education level in Spain.

The evolution of the Spanish education system since the Spanish Civil War (1936–1939) has not had a uniform impact on successive generations of Spanish students [11]. De la Fuente and Doménech [12] present a comprehensive description of the evolution in the educational attainment of the adult population in Spain between 1960 and 2010. They report a remarkable expansion in the average number of years of schooling in Spain, rising between 1991 and 2010 from 4.70 to 9.64 years. During their sample period, illiteracy was low in most developed countries; yet, Spain, along with other Southern European countries, continued to present significant rates. Remarkably, illiteracy in Spain over these past decades as good as disappeared, falling from 15.0% to 2.1%. Thus, the expansion of education in Spanish society over the last half-century rendered those with no formal education increasingly and negatively select, while the college-educated became less positively select. Since the 1960s, Spanish society has undergone major demographic, socioeconomic, and political change. Schooling, particularly under the conditions experienced in Spain, may have causally improved health, as over this period better access to such resources as more fulfilling jobs, greater economic security, stronger social ties, and healthier lifestyles was gained [13]. In 2019, in Spain, about 22% of the population aged 50 and over had a college education (calculation based on Spanish National Institute of Statistics (INE) data), indicating that as people get older this cohort becomes less select than in the past and the benefits of education extend among a larger number of people (Table S1).

### 1.2. Health Dynamics at Older Ages

Europeans live longer than ever and spend decades in retirement, but during a significant number of years at the end of their life course they have to live with disease and disability [5]. Surprisingly, Southern Europeans, who enjoy long LE at very old ages, do not appear to have better health and, indeed, often rank among the worst in terms of indicators of disability. For instance, Spaniards, leaders in terms of LE at birth [7], have some of the highest levels of functional problems [14].

These substantial increases in longevity were generally accompanied by improvements in the health of our societies, with a direct impact on survival, particularly, at older ages [15]. However, the aging process and the deterioration in health do not interact in the same way for all individuals. Indeed, a gender health–survival paradox exists whereby women experience a longer LE than men [16,17], but men’s health status tends to be better than that of their female counterparts [1].

### 1.3. Health and Education Inequalities

The association between education and longevity is one of the most consistent findings among social scientists. As noted, Jasilionis and Shkolnikov [10] showed that those with high levels of education generally have the highest LE and, moreover, lead the way towards a lengthening of life for the remaining population groups. Indeed, the most common findings are in line with this international overview with the highly educated being associated with longer, healthier lives than those with low levels of education who might experience higher mortality [6,18].

Cambois et al. [19] show that, in general, those with high levels of education have better health than those with low levels of education (and live more years in good health). Moreover, women with a low educational level appear to be the ones with the worst health [20].

In short, it seems vital that we examine educational differences in HLE to understand inequalities in general and, more specifically, to determine what gender patterns across educational attainment groups tell us. Thus, the aim of this study is to document the corresponding social disparities at ages 50-plus in a Spanish data set using different health indicators to quantify these differences across educational attainment groups.

## 2. Materials and Methods

### 2.1. Data

We draw on three sources of data maintained by the Spanish National Institute of Statistics (INE). First, our mortality data are taken from the 2012 Spanish National Registry of deaths by sex, age group, and level of educational attainment (available since 2016) [21]. The INE used a matching algorithm to link registered deaths to population databases and succeeded in assigning the level of education in 97 percent of deaths.

Second, to compute the population, we use INE records for five-year age groups by gender in 2012. We also consider the institutionalized population (i.e., in hospitals or nursing homes) obtained from the 2011 Population and Housing Census (Censos de Población).

Finally, we obtain the prevalence of each of our three health indicators (see below) using microdata from the 2011–2012 Spanish National Health Survey (ENSE).

### 2.2. Measures

We employ three indicators of health status to compute HLEs: (1) self-perceived health over previous 12 months, dichotomized into poor (regular, bad, and very bad) vs. good health (very good and good); (2) the global activity limitation indicator (GALI) measuring responses to the question, “For at least the past 6 months, to what extent have you been limited due to a health problem to perform the activities that people usually do?”, where those declaring themselves to be “Limited, but not severely” or “Severely limited” were assigned a value of 1 and those reporting no limitations were assigned a value of 0; and (3) reports on the presence (“having a chronic disease or health problem”) or absence of any diseases (“no health problems”) over the previous 6 months or more.

We classify our respondents into four groups based on years of education completed in accordance with the National Classification of Education 2000 (CNED-2000) [22]: (1) “primary education,” those with primary education or less and the illiterate; (2) “lower secondary education,” those with compulsory secondary education; (3) “upper secondary education,” those with noncompulsory secondary or medium professional education; and (4) “high education,” those with high professional education, university degrees of all levels, and PhDs (see Table S2 in the Supplementary Material).

### 2.3. Method

We first compute the crude and standardized mortality rates for men and women by level of education in 2012. The latter is calculated by applying a direct method for 2012 using the total Spanish population as reference. We then calculate the odds ratios (ORs) resulting from a logistic regression (not weighted) to evaluate the significant association adjusted by age between being female (male as reference) and level of education (primary education as reference) and the likelihood of having bad health.

We estimate LE by level of education by sex using conventional life tables and their confidence intervals with Chiang’s method [23]. Age is top-coded in a large age group at 85-plus; therefore, our abridged life table closes up to this age. Our two data sources—mortality rates by level of education from INE and prevalence of health problems by level of education from ENSE—are combined using Sullivan’s method to estimate HLE and unhealthy life expectancy (ULE) at 50 and we divide total LE into the expected number of years of life to be spent in a healthy or unhealthy state. Confidence intervals are provided using the tool developed by the European Health Monitoring Unit, including the institutionalized population and considering the variance for health variables. All data sources used in this analysis correspond to 2012 statistics, except for institutionalized population (2011, i.e., the census year) and the health survey (2011–2012).

## 3. Results

Table 1 shows the Spanish LE and mortality rates (crude and standardized) at age 50 for men and women by education level. Spanish LE at age 50 in 2012 was 31.1 and 36.5 years for men and women, respectively. In general, mortality rates are higher for men than for women, the same pattern being seen when the rates are standardized (29.2 and 18.3 for men and women, respectively). Generally, the higher the level of education, the lower the standardized mortality rates, which translates into a gradual increase in LE at age 50 as educational attainment increases. Spanish population with only primary education experienced the highest standardized mortality rates compared to all other education groups. Differences in standardized mortality rates are all statistically significant except for those for women between upper secondary and high education, where no such differences are found.

Spanish LE at age 50 varies substantially for men and women according to level of education. As expected, Spanish women live longer than men in all education groups. At 50, Spanish men (women) with primary education are expected to live 29.8 (35.9) vs. 33.6 (38.6) years for those with high education, a difference of 3.8 (2.7) years. Note the smaller gradient in the case of women.

Table 2 presents the age-adjusted prevalence of negative health measures by gender and the ORs for poor health indicating the effect of being female (male as reference) and education levels (primary education as reference) for men and women at age 50, adjusted by age. According to the health indicators, a higher proportion of females than males report poor self-perceived health (49% vs. 38%), GALI limitations (35% vs. 23%) and suffer from chronic diseases (64% vs. 58%). Controlling for age, the relative ORs for each of these health indicators in females are 1.59, 1.70, and 1.36, respectively.

Across educational groups, for both men and women, the prevalence of the three health measures falls as the level of education increases. In general, the relative ORs are statistically significant across education groups (primary education as reference) for the three health measures, except for some categories of the chronic diseases (secondary for men and lower secondary education for women).

Finally, Table 3 shows HLE (in years) and ULE (in years and as a percentage) at age 50 for each health indicator by gender across education groups. There are no substantial gender differences in terms of HLE; however, for all health measures, the HLE of the men is always higher than that of their female counterparts. In contrast, differences in unhealthy LE are substantial. For all health indicators, the proportion of life spent in an unhealthy state is higher for women than it is for men, ranging from 40.4 percent for GALI limitations to 69.2 percent for chronic diseases (27.3% to 62.1% for men, respectively).

An examination of the differences in healthy life years by education level reveals substantial differences in the numbers of years an individual can expect to live in good health, as well as differences by gender. As expected, the number of years lived in a healthy state rises notably with an increase in education level across all three health measures. However, the magnitude of these differences differs markedly by sex, being much greater for females than for males. For instance, when using self-perceived health to compute healthy life years, we see the largest education inequalities (8.7 vs. 11.6 years for men and women, respectively). In contrast, when measuring healthy life years in terms of chronic diseases, we find the smallest gender and education gaps.

The outcomes change somewhat, however, when we consider the percentage of years lived in an unhealthy state by gender across education level for each indicator. Females can expect to spend more years in poor health than males for all health indicators, while the largest education gap for both genders is found for self-perceived health. In general, those with a high level of education live fewer years in poor health, except in the case of men where there is no educational gradient for chronic diseases. Thus, the proportion of years of life lived with chronic diseases is roughly the same for men with low levels of education as it is for men with high levels (62.1% vs. 60.1%).

## 4. Discussion

Our findings highlight the importance of the level of education achieved in extending the proportion of healthy life years and improving quality of life in a Southern European country. Among the 50-plus, a low level of education not only translates into a lower LE, but it also means spending less time in good health and suffering more years of poor health. Our findings confirm that Spanish women have a longer LE at age 50, but experience a higher number of additional years in poor health and a lower percentage of healthy years than men, without exception, across the levels of education.

Gender differences are marked across educational groups. The highest gender differences in LE are reported between those with primary and upper secondary educations and the lowest between those with the highest level of education. However, the LE advantage of women disappears when we consider our health controls and compute HLE.

We should also highlight the importance of self-perceived health among men and women. This indicator presents the highest educational gap for both men and women, suggesting that when a subjective health measure is used, the variation across education groups is at its greatest.

Although we detect differences in LE by gender and level of education, the LE of males with high levels of education is lower than that of the females even with the lowest levels of education. These results allow us to reconstruct a perfect gradient beginning with men with low levels of education through to women with high levels of education. The larger gradient for self-perceived health compared to that for chronic conditions was also reported elsewhere [24].

Our study contributes to the current debate on improving the quality of life in good health given that, for the first time, data on educational inequalities in Spain are presented that combine mortality and morbidity rates. These findings are in line with outcomes reported in international literature [25]; however, these fresh Spanish data add new knowledge to the study of contextual factors as the determinants of social inequalities. Studies of mortality differentials in Spain showed fewer inequalities [26], but until now it was impossible to analyze these social differentials using HLE. Thus, Spanish data should make an important contribution to our understanding of the determinants of health inequalities and provide elements for intervention in the improvement of the population’s health. Indeed, our initial findings appear to show that the socioeconomic gradients in HLE are lower in Spain than those in Nordic countries, such as Denmark [27]. Likewise, other comparative studies drawing on data for the Spanish regions [28] confirm that these gradients present some of the smallest differences in HLE in Europe. Yet, although Spain presents common, robust evidence of its mortality advantage over most European countries, this evidence is less clear when HLE inequalities are considered.

Following on from this article, our future research lines will investigate educational trends over time, to examine whether these inequalities persist or whether we see a convergence by level of education and gender. Overall, achieving a healthier society should reduce pressure on the public health system, while identifying the most deprived groups—putatively those with the lowest education level—should help policymakers design appropriate plans of action.

A number of potential limitations that might affect the results described above should be mentioned. First, our study employs cross-sectional data and, second, different data sources. The latter leave our results open to the possible risks associated with the process of imputation of the level of education to the mortality data, performed for the first time by the INE in 2012 (the year of our study). However, the low percentage of unallocated records and the methodological guarantees provided by the official statistics ensure the quality of the data used. Third, the heterogeneity in existing methods limits the comparison of our findings with other studies examining health expectancies [28]. Finally, a further limitation might derive from the characteristics of the indicator of social position used, i.e., the level of education. Education serves here as our indicator of social inequality for mainly two reasons. First, we argue that, unlike a person’s occupation, education is more likely to remain constant throughout their adult life, at least for most people. And second, level of education, which is typically well reported in questionnaires and survey data, is strongly related to working conditions and income. However, level of education does present a series of limitations, especially when we want to make comparisons between countries or over time, since the precise meaning of different educational achievements might vary between countries [29]. Thus, for Spain, as for the rest of Southern Europe, due to the delay in the process of modernization, education levels might be a less important element of social stratification than it is in places with more advanced processes, particularly at older ages. This could lead to an underestimation of health inequalities in these Southern European countries and account for part of the lower inequality found [26].

## 5. Conclusions

Overall, our study describes notable inequalities both in the duration of life and in the quality of life of Spain’s older generation. The results have important implications for health policies, as well as those aimed at promoting active aging and a good quality of life. The strong association between education and overall population health, particularly in Spain over the last 60 years, suggests that education policies may also be seen as indirect health policies. Hence, the design of prospective public health policies should pay special attention to the least-educated individuals, who represent approximately 35.7% of males and 47.0% of females aged 50-plus in Spain in 2012. Thus, primary education is a sector that should attract public investment in order to improve health in Spain. Our study also provides new evidence of a possibly lower gradient in mortality and health in a Southern European country with higher levels of social inequality and a limited social protection system. Although this lower gradient may, in part, be explained by a later process of economic modernization, it might also be explained by the existence of health assets that have traditionally received less attention in these countries. These health assets, such as greater family network density and less inequality of access to healthy food, can be critical elements of political action. Indeed, an effective health policy needs to incorporate a positive vision of these assets and recognize the importance for a population’s health of factors that lie outside what might strictly be considered the field of health. This opens up new research hypotheses that need to be further explored and which, doubtless, will provide important evidence favoring the implementation of fairer and healthier policies.

## Figures and Tables

**Table 1:** Life expectancy, crude, and standardized mortality rates at age 50 by gender and educational level.

Gender and Education Level	Life Expectancy	CI (95%)	Crude Mortality Rate	Standardized Mortality Rate	CI (95%)
Men	31.1	(31.05–31.14)	25.08	29.23	(29.11–29.35)
Women	36.5	(36.40–36.53)	21.16	18.34	(18.25–18.42)
Gender Gap	5.4		−3.92	−10.89	
**Men**					
Primary Education	29.8	(29.74–29.91)	40.52	31.02	(30.82–31.22)
Secondary Education					
Lower	31.8	(31.69–31.96)	16.96	27.22	(27.00–27.43)
Upper	32.4	(32.17–35.61)	13.92	25.82	(25.52–26.12)
High Education	33.6	(33.41–33.80)	13.1	23.6	(23.26–23.84)
Education Gradient (high vs. primary)	3.8		−27.42	−7.42	
**Women**					
Primary Education	35.9	(35.85–36.01)	32.95	19.18	(19.05–19.31)
Secondary Education					
Lower	37.5	(37.41–37.73)	11.95	16.47	(16.32–16.63)
Upper	38.5	(38.20–38.82)	8.67	15.01	(14.78–15.24)
High Education	38.6	(38.31–38.93)	7.62	14.86	(14.64–15.08)
Education gradient (high vs. primary)	2.7		−25.33	−4.32	

Source: Spanish National Registry of deaths and population records, Spanish National Institute of Statistics (INE).

**Table 2:** Age-adjusted prevalence of reporting bad health (three health status measures) for men and women by education level. GALI is the global activity limitation indicator; odds ratios (OR) for poor health indicates the effect of being female and education level for men and women, adjusted by age (for age 50).

	Bad Self-Perceived Health			GALI (Being Limited)			Chronic Disease		
	Percentage	OR	CI (95%)	Percentage	OR	CI (95%)	Percentage	OR	CI (95%)
**Men**	37.5	1		23.3	1		57.7	1	
**Women**	48.8	1.59	(1.47–1.72)	34.8	1.70	(1.56–1.85)	64.1	1.36	(1.26–1.48)
**Gender Gap**	11.3			11.6			6.4		
**Men**									
Primary Education	49.6	1		30.4	1		62.9	1	
Secondary Education									
Lower	39.6	0.78	(0.69–0.89)	23.1	0.83	(0.72–0.97)	61.0	0.99	(0.87–1.13)
Upper	29.5	0.5	(0.43–0.58)	20.1	0.68	(0.57–0.81)	58.4	0.88	(0.77–1.02)
High Education	17.5	0.4	(0.34–0.46)	12.4	0.57	(0.48–0.68)	42.5	0.86	(0.75–0.99)
**Education gradient** (high vs. primary)	−32.1			−18.0			−20.4		
**Women**									
Primary Education	60.2	1		43.2	1		69.3	1	
Secondary Education									
Lower	50.2	0.67	(0.60–0.75)	32.7	0.74	(0.66–0.82)	68.2	0.9	(0.81–1.01)
Upper	36.3	0.45	(0.39–0.51)	28.6	0.64	(0.55–0.74)	63.3	0.79	(0.70–0.91)
High Education	16.6	0.28	(0.24–0.32)	15.4	0.52	(0.45–0.61)	37.3	0.64	(0.56–0.73)
**Education gradient** (high vs. primary)	−43.6			−27.8			−32.0		

Source: Spanish National Registry of deaths and population records, Spanish National Health Survey (ENSE) and Population and Housing Census, INE.

**Table 3:** Healthy life expectancy and unhealthy life expectancy (ULE), years, and percent in each health indicator by gender and education level for ages 50-plus.

	Self-Perceived Health			GALI			Chronic Disease		
	HLE	ULE		HLE	ULE		HLE	ULE	
		Years	%		Years	%		Years	%
**Men**	18	13.1	42.1	22.6	8.5	27.3	11.8	19.3	62.1
	(17.6–18.5)	(12.6–13.5)		(22.2–23.0)	(8.1–8.9)		(11.4–12.2)	(18.9–19.7)	
**Women**	16.7	19.7	54.1	21.7	14.7	40.4	11.2	25.2	69.2
	(16.3–17.2)	(19.3–20.1)		(21.3–22.2)	(14.3–15.1)		(10.8–11.6)	(24.8–25.6)	
**Gender Gap**	−1.3	6.6	12.0	−0.9	6.2	13.1	−0.6	5.9	7.2
**Men**									
**Primary education**	15.1	14.8	49.5	20.7	9.2	30.7	11.3	18.5	62.1
	(14.3–15.8)	(14.1–15.5)		(20.0–21.3)	(8.5–9.8)		(10.6–12.1)	(17.8–19.2)	
**Secondary Education**									
**Lower**	18	13.8	43.4	23.5	8.3	26.1	11.5	20.4	63.9
	(17.1–18.9)	(12.9–14.8)		(22.7–24.4)	(7.4–9.1)		(10.6–12.3)	(19.5–21.2)	
**Upper**	20.8	11.6	35.8	23.8	8.6	26.5	12.7	19.7	60.8
	(19.3–22.2)	(10.2–13.1)		(22.5–25.1)	(7.3–9.9)		(11.3–14.1)	(18.3–21.1)	
**High education**	23.8	9.8	29.2	25.6	8.0	23.7	13.4	20.2	60.1
	(22.5–25.1)	(8.5–11.1)		(24.4–26.9)	(6.7–9.2)		(12.1–14.7)	(18.9–21.5)	
**Education gradient** (high vs. primary)	8.7	−5.0	20.3	4.9	−1.2	−6.9	2.1	1.7	−2.0
**Women**									
**Primary Education**	13.4	22.6	62.8	19.5	16.5	45.8	9.8	26.1	72.7
	(12.7–14.0)	(21.9–23.3)		(18.8–20.1)	(15.8–17.1)		(9.2–10.5)	(25.5–26.8)	
**Secondary Education**									
**Lower**	18	19.6	52.1	23.3	14.3	38.0	11.3	26.3	69.9
	(17.1–18.9)	(18.7–20.4)		(22.4–24.1)	(13.5–15.1)		(10.5–12.0)	(25.5–27.1)	
**Upper**	22.3	16.2	42.1	25.6	12.9	33.5	11.4	27.1	70.4
	(20.5–24.1)	(14.4–18.0)		(23.9–27.4)	(11.1–14.7)		(10.0–12.7)	(25.8–28.5)	
**High education**	25	13.7	35.4	25.7	12.9	33.4	15.2	23.4	60.6
	(23.2–26.7)	(11.9–15.4)		(23.9–27.5)	(11.1–14.8)		(13.6–16.8)	(21.8–25.0)	
**Education gradient** (high vs. primary)	11.6	−8.9	−27.4	6.2	−3.6	−12.4	5.4	−2.7	−12.1

Source: Spanish National Registry of deaths and population records, ENSE, and Population and Housing Census, INE. Note: Life expectancy and healthy life expectancy (HLE) are computed with 95% confidence intervals (in parentheses).

## Supplementary Materials

The following are available online at https://www.mdpi.com/1660-4601/17/10/3558/s1, Table S1: Proportion (%) of people by level of education and 5-years age group for men and women, 2012, Table S2. Comparison between the International Standard Classification of Education and the classification used in our analysis.
