# Supplementary material for: Educational Inequalities in Life and Healthy Life Expectancies among the 50-Plus in Spain
Source: Int J Environ Res Public Health. 2020 May 19;17(10):3558. doi: 10.3390/ijerph17103558 (PMC7277913; doi:10.3390/ijerph17103558)
Supplement: Supplementary file 1 [file ijerph-17-03558-s001.pdf]

## Supplementary Material

**Table S1.** Proportion (%) of people by level of education and 5-years age group for men and women, 2012.

| Age groups | Primary Education | Secondary Education | | High education |
|---|---|---|---|---|
| | | Lower | Upper | |
| | | Men (%) | | |
| 50-54 | 17.5 | 33.9 | 21.6 | 26.9 |
| 55-59 | 22.4 | 34.4 | 18.9 | 24.3 |
| 60-64 | 31.5 | 32.3 | 14.8 | 21.4 |
| 65-69 | 40.8 | 29.1 | 11.7 | 18.5 |
| 70-74 | 51.5 | 23.9 | 9.7 | 14.9 |
| 75-79 | 61.4 | 19.5 | 7.6 | 11.6 |
| 80-84 | 67.2 | 16.2 | 6.5 | 10.1 |
| 85+ | 69.6 | 14.8 | 6.3 | 9.4 |
| Total | 37.5 | 28.5 | 14.3 | 19.7 |
| | | Women (%) | | |
| 50-54 | 18.7 | 34.4 | 21.6 | 25.3 |
| 55-59 | 26.5 | 36.4 | 17.6 | 19.4 |
| 60-64 | 38.1 | 34.9 | 12.8 | 14.1 |
| 65-69 | 50.5 | 29.7 | 9.3 | 10.5 |
| 70-74 | 61.6 | 23.4 | 7.4 | 7.6 |
| 75-79 | 70.2 | 18.7 | 5.5 | 5.6 |
| 80-84 | 74.4 | 15.9 | 4.9 | 4.8 |
| 85+ | 76.8 | 14.4 | 4.5 | 4.2 |
| Total | 47.0 | 27.9 | 11.9 | 13.2 |

Source: Spanish Population register, 2012.

**Table 2.** Comparison between the International Standard Classification of Education and the classification used in our analysis.

| ISCED Classification | | Our Classification | | |
|---|---|---|---|---|
| **Education groups** | **ISCED** | **Education groups** | | |
| **LOW** | 0 pre-primary education<br>1 primary education | **Primary Education** | | 01Analfabetos<br>02Estudios primarios incompletos<br>03Educación primaria |
| **MIDDLE** | 2 lower secondary education<br>3 (upper) secondary education<br>4 post-secondary non tertiary education | **Secondary Education** | **Lower** | 04Primera etapa de educación secundaria y similar |
| | | | **Upper** | 05Segunda etapa de educación secundaria con orientación general<br>06Segunda etapa de educación secundaria con orientación profesional<br>07Educación postsecundaria no superior |
| **HIGH** | 5 first stage of tertiary education (not leading directly to an advanced research qualification)<br>6 second stage of tertiary education (leading to an advanced research qualification) | **High Education** | | 08Enseñanzas de formación profesional, artes plásticas y diseño y deportivas de grado superior y equivalentes; títulos propios universitarios que precisan del título de bachiller, de duración igual o superior a 2 años<br>09Grados universitarios de 240 créditos ECTS, diplomados universitarios, títulos propios universitarios de experto o especialista, y similares<br>10Grados universitarios de más de 240 créditos ECTS, licenciados, másteres y especialidades en Ciencias de la Salud por el sistema de residencia, y similares<br>11Másteres, especialidades en Ciencias de la Salud por el sistema de residencia y similares<br>12Doctorado universitario |
